# Changing the default for tobacco-cessation treatment in an inpatient setting: study protocol of a randomized controlled trial

**DOI:** 10.1186/s13063-017-2119-9

**Published:** 2017-08-14

**Authors:** Babalola Faseru, Edward F. Ellerbeck, Delwyn Catley, Byron J. Gajewski, Taneisha S. Scheuermann, Theresa I. Shireman, Laura M. Mussulman, Niaman Nazir, Terry Bush, Kimber P. Richter

**Affiliations:** 1 Department of Preventive Medicine and Public Health, University of Kansas Medical Center, 3901 Rainbow Boulevard, Kansas City, KS 66160 USA; 2 University of Kansas Cancer Center, 3901 Rainbow Boulevard, Kansas City, KS USA; 3 Children’s Mercy Hospitals and Clinics, Center for Children’s Healthy Lifestyles and Nutrition, Kansas City, MO USA; 4 Brown University, Providence, RI USA; 5 Optum, Seattle, WA USA

**Keywords:** Tobacco use disorder, Smoking cessation, Hospital, Randomized clinical trial, Motivation, Treatment guidelines

## Abstract

**Background:**

Most health care providers do not treat tobacco dependence routinely. This may in part be due to the treatment “default.” Current treatment guidelines recommend that providers (1) ask patients if they are willing to quit and (2) provide cessation-focused medications and counseling only to smokers who state that they are willing to quit. The default is that patients have to “opt in” to receive cessation assistance: providers ask smokers if they are willing to quit, and only offer medications and cessation support to those who say “yes.” This drastically limits the reach of cessation services because, at any given encounter, only one in three smokers say that they are ready to quit. The objective of this study is to determine the impact of providing all smokers with tobacco-cessation treatment unless they refuse it (OPT OUT) versus current practice—screening for readiness and only offering treatment to smokers who say they are ready to quit (OPT IN).

**Methods:**

This individually randomized clinical trial is conducted in a tertiary-care hospital. We will conduct the trial among up to 1000 randomly selected hospitalized smokers to determine the population impact of changing the treatment default, identify mediators of outcome, and determine the cost-effectiveness of this new, highly proactive approach. This is a population-based study that targets an endpoint of vital interest; applies minimal eligibility criteria to broaden generalizability; and utilizes hospital staff for interventions to ensure long-term sustainability. The study employs delayed consent and an innovative Bayesian adaptive design to evaluate a major shift in our approach to care. If effective, this change would expand the reach of tobacco-cessation treatment from 30% to 100% of smokers.

**Discussion:**

Regardless of outcome, the trial will provide a model of how to alter and evaluate the impact of health care defaults. If OPT OUT proves to be more effective, it will expand the population eligible for cessation treatment by over 300%. It will also simplify the tobacco-cessation treatment algorithm, and relieve busy health care providers of the burden of evaluating readiness to quit.

**Trial registration:**

Clinical Trials Registration, ID: NCT02721082. Registered on 22 March 2016.

**Electronic supplementary material:**

The online version of this article (doi:10.1186/s13063-017-2119-9) contains supplementary material, which is available to authorized users.

## Background

Based on current rates of tobacco use uptake and cessation, 20 million Americans will die from tobacco-related illnesses between 2000 and 2050 [1]. Due to the 20–40-year time lag between starting smoking and the onset of tobacco-related illnesses [2], helping smokers quit is the best way to immediately reduce illness and deaths. Guideline-based tobacco-cessation treatment consists of two branching approaches [3]: motivational counseling, based on the principles of Motivational Interviewing [4], for patients not willing to make a quit attempt; and cessation-oriented pharmacotherapy and counseling for smokers who are prepared to make a quit attempt. Motivational treatment focuses on building smokers’ motivation to quit, but its effectiveness for smoking cessation is, at best, mixed [5].

Cessation-oriented pharmacotherapy and counseling double quit rates over no-treatment controls [3, 6], but the “default option” for patients unwilling to quit tobacco use is “no treatment.” With each step of guideline-based tobacco-cessation treatment, smokers must say “yes” (*are they willing to quit, do they want medications, do they want counseling*) in order to receive evidence based care—they must “opt in.” This is in stark contrast to treatment of other common medical conditions, such as hypertension, where the default option is “treatment”: physicians identify and initiate treatment for hypertension [7], and patients must “opt out” if they do not want care.

Likely as a result of this tobacco-cessation treatment “default,” fewer than one in five smokers actually get assistance in quitting on any given outpatient encounter with their health care providers [8–10]. In hospitals, an even smaller percentage of smokers receive assistance: a meta-analysis found that only 14% of inpatient smokers were provided with a prescription for cessation medication and 12% received referrals for follow-up [11]. Moreover, because smokers must opt in for cessation counseling and medication separately, few receive both components of evidence-based care [12]. Many would blame this treatment gap on smokers and their lack of motivation to quit smoking. Another potential cause, however, is how the “default” treatment for tobacco dependence is currently constructed in US health care systems.

The treatment default for tobacco is different from any other chronic health condition. In health care, most treatment guidelines direct clinicians to provide evidence-based treatment, which the patient will receive by default unless they refuse treatment. In fact, this choice architecture is arguably the most ethical. Where there is strong clinical evidence that supports an appropriate therapy, the therapy should be presented as the default [13]. Hence, the exceptional position that smokers should be asked if they are “willing” to quit creates a rate-limiting step in tobacco-cessation treatment that may be less effective and less ethical than an opt-out approach. Details on how defaults potentially affect treatment choices, and how this relates to tobacco-cessation treatment, may be found in a commentary by Richter and Ellerbeck [14].

Decision theorists suggest that institutions should structure default choices to be the options that make the choosers better off, as judged by themselves [15]. Tobacco-cessation treatment is an excellent candidate for a default that favors treatment because 70% of smokers state that they ultimately want to quit smoking [12]. Choosing to quit in the near future, however, is extremely difficult for smokers because they get the pleasures of smoking, and the pain of abstaining from smoking in the present, but suffer the terrible consequences of smoking in the future [16]. This may be why fewer smokers state that they are ready to quit in the near future. They could use a “nudge” to accept treatment in order to reach their ultimate goal of quitting. In our opinion, smokers fail to receive effective cessation treatment due to the way in which the US structures the tobacco-cessation treatment default [14]. In this study, we will examine the effects of proactively providing cessation-oriented treatment to all smokers, regardless of their willingness or readiness to quit.

The objective of the study is to determine the impact of providing all smokers with cessation pharmacotherapy and counseling unless they refuse it (OPT OUT) versus current practice—screening for readiness and only offering cessation assistance to smokers who say they are ready to quit (OPT IN).

## Methods

### Design and setting

**Fig. 1:**
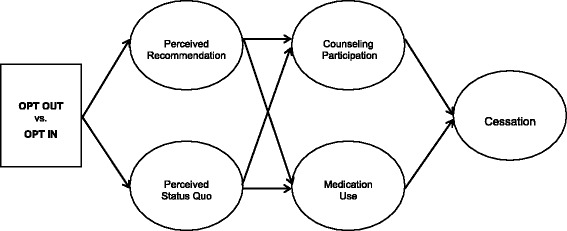
Theoretical model

The study is a prospective, randomized, comparative effectiveness study using a Bayesian adaptive design. It is a two-arm study with individual randomization to groups in a large Midwestern academic medical center (Fig. 1). The hospital is a 475-bed tertiary-care hospital that admits over 20,000 patients per year and is located in a large metropolitan area. The study was approved by the University of Kansas Human Subjects Committee (IRB00006196; STUDY00001774)

### Study procedures

The study employs a modified Zelen design for consent [17, 18]. Study staff will select, randomize, and intervene with patients prior to collecting verbal consent at the 1-month post-discharge visit. For the outcome analysis, the study uses a Bayesian adaptive trial design [19]. Mediation analyses will also provide insight into why changing the default did, or did not, work. We hypothesize that smoker perceptions of what course of action their cessation counselor recommends, and what is the status quo for receiving medications and counseling at the hospital, will affect their counseling participation, medication use, and quit rates (Fig. 1). The trial will be implemented in four stages over 4.5 years (Table 1).

**Table 1:** Clinical trial timeline

Month	Stage	Milestones (by end of year, unless otherwise stated)
6–23	1: Early implementation	• 400 participants consented • 6-month outcome data collected on 200 participants • Manuscript on study design under review
24–39	2: Implementation	• Baseline data collected on 800 participants • 6-month data collected on 600 of participants • Data cleaned and prepared for analysis on half of all participants
40–51	3: Early analyses	• Complete consent of final 200 participants by midyear • 6-month data collection completed on all participants • Manuscripts on baseline characteristics of participants published
52–60	4: Analysis/dissemination	• All data cleaned and prepared for analysis • Conduct all outcome, mediator/moderator analyses • Manuscripts on study outcomes under review

### Recruitment of study participants

#### Random selection for the trial via the electronic health record (EHR)

Study staff have access via the EHR to a real-time, comprehensive list of all smokers in the hospital—the *tobacco use list*. For the purposes of the study, research staff will randomly select patients from the tobacco use list, and provide selected patients’ names to study staff for baseline assessment, randomization, and intervention. Random selection of participants serves two purposes. First, it ensures that we will test the change of default among a sample representative of all hospitalized smokers, which will enhance the generalizability of the findings. Second, it ensures that smokers *not* seeking tobacco-cessation treatment will be included in the trial, which will enhance our ability to detect the effect of changing the default among smokers who have not requested, and who might not be considered “ready” for medications and counseling.

#### Patients excluded from random selection via EHR

A number of hospital services routinely do not permit a majority of their patients to be placed on Nicotine Replacement Therapy (NRT). These include the operating room, orthopedic surgery, gastro-intestinal surgery, the burns service, and the neurology stroke service. Patients on these units, as well as patients less than 18 years of age, patients who have been in the hospital longer than 3 days, and pregnant patients, will be excluded from the tobacco use list prior to random selection for the trial. Patients who requested, or have orders for, tobacco-cessation treatment will be treated as usual by the existing hospital tobacco-cessation treatment service and will only be included in the trial if randomly selected.

#### Eligibility and baseline assessment

Study staff will visit all randomly selected smokers. For all study patients, staff will assess and address patient comfort, provide brief advice to quit, and provide a quit-smoking pamphlet. Staff will then assess eligibility and collect baseline data. Initial study eligibility criteria include: (1) the ability to speak English or Spanish, (2) have no significant comorbidity that precludes participation (i.e., acute, life-threatening illness or altered mental status such as dementia), (3) be a permanent resident of the state of Kansas or Missouri, (4) provide a secondary telephone contact to ensure 1-month follow-up survey completion, and (5) completed all eligibility questions.

Other eligibility criteria ensure that patients are able to benefit from the intervention. Because follow-up counseling is by telephone, participants are required to have access to a telephone or mobile phone. Because patients are receiving free starter packs of NRT and prescriptions for post-discharge smoking cessation medications, eligibility is limited to patients who smoke one or more cigarettes per day, have smoked at least 25 of the past 30 days, and are otherwise medically eligible to use NRT (i.e., no acute myocardial infarction/ST-elevation myocardial infarction (STEMI), or other acute heart conditions).

Last, several criteria preserve our ability to detect the effects of the trial. Patients cannot already have been seen by hospital tobacco-cessation treatment (UKanQuit) counselors or study staff during the recruitment hospitalization, cannot currently be taking cessation medications, or cannot be enrolled in a separate quit-smoking program.

Study staff will record eligibility for the study on their clinic service tablet computer, along with standard service administrative data, which includes demographics, smoking characteristics, readiness to quit, and contact information for the 1-month follow-up. These administrative data will constitute baseline data for the clinical trial.

The patients who are not eligible will be provided with the typical hospital tobacco-cessation services but will not be enrolled into the trial. The typical hospital services will follow the guidelines of UKanQuit [20], which is a dedicated tobacco-cessation treatment service funded by the hospital. UKanQuit services include: (1) working with the medical team to address inpatient withdrawal symptoms, usually with NRT, (2) assessing readiness to quit, (3) providing motivational counseling to smokers who are not ready to quit, and (4) arranging for post-discharge counseling and medication prescriptions for patients who are ready to quit. The principal investigator (PI) of the present study also directs UKanQuit, and staff for both projects will work together closely to ensure that the study and the UKanQuit service avoid duplicating intervention among the same patients.

#### Enrollment and random allocation

All patients who are eligible will be automatically enrolled into the trial and randomly allocated to a treatment arm. Consent for the trial is delayed—it is sought at the 1-month follow-up. A function will be programmed into the tablet intake form so that study staff will select a key to randomize eligible smokers to either OPT OUT or OPT IN. At the beginning of the trial, the function will assign patients to groups in a 1:1 allocation ratio. Later, based on intermediate outcomes, the study biostatistician may alter the ratio in accordance with the Bayesian study design (see “Data analysis,” below). Study staff will offer smokers motivational counseling, medications, and cessation-oriented practical counseling in accordance with the study arm to which patients are randomized. Patients are only randomized into our trial once. If someone has been randomized into the trial in the past—regardless of whether they provided consent at the 1-month follow-up or not—they are not eligible for repeat randomization. There are likely cases where a randomly selected patient is screened and found ineligible at one point in time, then eligible at a later readmission date. These patients may be enrolled at the later admission date.

#### Rationale for conducting a study with delayed consent

The study uses a modified Zelen design, in which consent is obtained after randomization and treatment. We considered consenting patients before randomization and treatment. This would, however, require patients to “opt in” to being in a trial and possibly exclude the very smokers who might benefit from changing the default. We rejected consent before randomization and treatment, as it is the very experimental manipulation we seek to test. The Zelen design, in which patients are consented after randomization, can markedly enhance recruitment and improve generalizability by including a higher proportion of eligible patients [21, 22]. In a departure from most traditional Zelen design trials, the proposed study will delay consent until after treatment in order to examine the impact of OPT OUT on cessation at 1-month post discharge.

#### OPT IN and OPT OUT intervention procedures—framing the default

We have created language that constitutes the “choice architecture” for each study condition (Table 2)*.* We have crafted these phrases to be short and simple, to enable study staff to reliably use them. Based on the patients’ group assignment, staff will frame the default and provide the appropriate intervention.

**Table 2:**
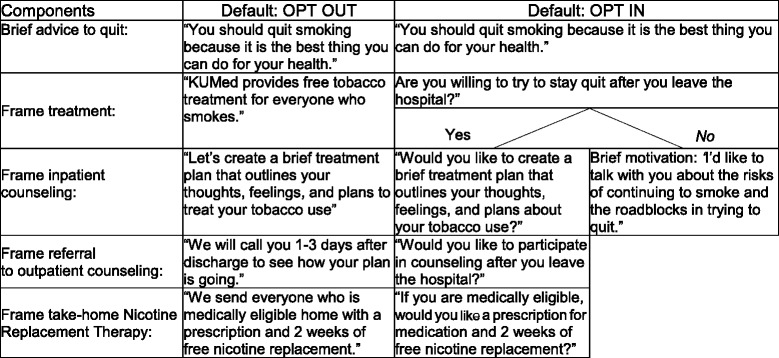
Treatment options by study arm

#### Inpatient counseling and treatment plan—OPT OUT

For all patients in OPT OUT, staff will provide brief practical counseling, complete a treatment plan, and provide a pamphlet that outlines tips on quitting. The treatment plan includes: (1) inpatient medications, (2) outpatient prescription for medication and a free “starter pack” of medications, and (3) post-discharge counseling. In describing the treatment plan for the patients, study staff operationalize constructs thought to underpin the power of the default [23]. These terms: (1) signal the provider’s positive attitude towards medication and counseling, and (2) state that the hospital’s status quo is to provide medications and counseling.

Using this OPT OUT language, staff will clearly indicate that they believe medications and counseling will benefit the patient, and that the hospital routinely and proactively provides cessation-oriented care to smokers. Unless a patient “opts out” of any or all elements of the treatment plan, all patients will receive all elements of the treatment plan, including inpatient medications, outpatient prescription and starter pack, and post-discharge counseling calls.

#### Inpatient counseling and treatment plan—OPT IN

For patients in OPT IN, staff will screen for willingness to stay having quit after leaving the hospital. Patients who are willing to quit will be offered similar counseling, treatment planning, and medications as patients in OPT OUT, including a pamphlet with tips on quitting. However, instead of proactively providing these services, staff will ask patients if they would like each element of the treatment plan—post-discharge counseling and/or post-discharge prescription for medication. Patients will only receive the elements of care to which they opt in.

Patients in OPT IN who are not willing to quit will receive brief intervention, in accordance with current treatment guidelines. This consists of a four-page pamphlet with resources for quitting and a brief counseling session that addresses the “5 Rs” of motivational counseling (*Relevance, Risks, Rewards, Roadblocks, Repetition*) [3].

#### Post-discharge treatment—OPT OUT—counseling

All patients in OPT OUT will be enrolled in post-discharge counseling that will be delivered by study staff. Participants who accept enrollment into counseling services will receive up to four proactive counseling calls in each of the 4 weeks following discharge. Each call is designed to provide practical counseling to help participants develop problem-solving and coping skills, secure social support, and design a plan for successful cessation and long-term abstinence. Initial calls last approximately 30 min and follow-up calls last on average 15 min. Once participants quit smoking, counselors review high-risk situations, coping skills, and stress management to prevent relapse. When participants “slip,” counselors troubleshoot relapse situations and encourage smokers to quit again. We will use RedCAP to store data for all calls, including number of attempts to reach the smoker, number of calls completed, and duration of calls.

#### Post-discharge treatment—OPT OUT—medication prescription and NRT “starter pack”

Study staff will work with all patients in OPT OUT to select a long-term cessation medication and plan how they will obtain and fill prescriptions post discharge. Study staff will request a prescription for post-discharge cessation medication via a note in the patients’ medical record, and via a text message to the floor pharmacist assigned to the patients’ medical team. The patients’ hospital physician will make the final determination regarding whether to write a cessation medication prescription, and which medication to prescribe.

To ensure that patients leave the hospital with some form of cessation medication, in order to avoid relapse, study staff will provide all OPT-OUT patients with a 2-week starter pack of over-the-counter quit-smoking medications. This will include 14 days of combination nicotine replacement pharmacotherapy, consisting of 14 nicotine patches plus 14-day supplies of either (a) nicotine gum, or (b) nicotine lozenges. The choice of the short-acting NRT will be made based on contraindications, past history of success/failure, and personal preferences [3]. On the day of hospital discharge, study staff will provide the sealed starter pack to the patient at the bedside, for the patient to use once they leave the hospital.

#### Post-discharge treatment—OPT IN—counseling and medications

Study staff will provide OPT-IN patients who “opted in” to post-discharge counseling the same counseling that is provided to OPT-OUT patients. Likewise, for OPT-IN patients who want a prescription for post-discharge medications, study staff will arrange for a prescription and provide a starter pack in the same manner as these elements are provided to OPT-OUT patients.

#### Month-1 call for service data collection and informed consent

In accordance with standard procedures in the existing UKanQuit treatment program [20, 24], study staff will call all patients at 1 month post enrollment to assess outcomes including smoking status, quit attempts, counseling utilization, medication use, and other factors related to quitting (see Table 3, Core study measures). At the close of the call, study staff will verbally debrief patients on their inclusion in the trial and invite them to participate in the study. Study staff will collect additional data related to study outcomes.

**Table 3:** Core study measures

	Baseline	Month 1	Month 6
Outcomes			
7-day point-prevalence abstinence		✓	✓
Biochemical quit verification		✓	✓
Number of quit attempts since enrollment		✓	✓
Post-discharge counseling adherence/other support		✓	✓
Medication use/adherence		✓	✓
Sociodemographics/mediators/moderators			
Demographics: age, gender, race	✓		
Length of hospital stay (for index visit)	✓		
Reason for hospitalization (index visit)	✓		
Readiness to quit, craving/withdrawal	✓	✓	
Number of cigarettes per day (cpd); time to first cigarette	✓	✓	
Motivation/confidence quit/stay having quit	✓	✓	
Default constructs (perceived status quo, etc.)		✓	
Rehospitalization		✓	✓
Cost measures			
Counseling		✓	✓
Nicotine Replacement Therapy (NRT) (calculated from patient self-reported use)		✓	✓

#### Rationale for measuring main outcome at 1 month post intervention

The present study is focused on how best to engage smokers in tobacco-cessation treatment (i.e., counseling and medications) and quit. Assessing outcomes at 1 month will best capture the immediate impact of OPT IN versus OPT OUT on medication/counseling use, quit attempts, and abstinence [25, 26]. Moreover, assessing outcomes at 1 month is in accordance with Joint Commission Guidelines and UKanQuit standard practice for post-discharge follow-up of hospitalized smokers [27]. Perhaps most importantly, 1 month represents an important timeframe for hospitals, as hospitals with excessive 1-month readmission rates for selected diagnoses will receive decreased Medicare reimbursements [28]. Should our intervention prove effective, it could pave the way for future studies on the impact of smoking cessation on reduced 30-day readmission rates for specific diagnoses. Those who refuse consent at 1 month will not be eligible for inclusion again into the trial and they will be included (simply counted as smokers, because we will have no data on them) in our simplified intent-to-treat (ITT) analyses.

#### Reimbursement

Patients who participate and complete the 1-month survey will be reimbursed US$25 whether or not they consent to participate in the clinical trial. Study staff will reimburse patients who consent to participating in the clinical trial and completing the extended 1-month survey with an additional US$25. All reimbursements will be via reloadable debit cards. The debit cards utilize the MasterCard payment system and are accepted at virtually every institution that accepts a credit card. Participants will also be reimbursed US$25 for the 6-month follow-up survey and US$50 for each salivary cotinine sample returned.

### Project measures (Table 3)

#### Tobacco abstinence

Outcome measures are adapted from the Society for Research on Nicotine and Tobacco’s Workgroup on Abstinence Measures and Workgroup on Biochemical Verification [29, 30]. Our primary endpoint is 7-day, verified cigarette abstinence at 1 month after enrollment. Subjects with missing data will be counted as smokers.

#### Verification of abstinence

We will use either mailed salivary cotinine or in-person carbon monoxide (CO) testing to confirm smoking status. Participants who report 7-day point-prevalence abstinence, and who are not taking NRT, will be asked to provide a saliva sample. Cotinine is the measure of choice because of its sensitivity and specificity [30]. Samples will be stored in a − 20 °C freezer until laboratory analysis. Participants who are still using NRT, or who refuse salivary cotinine, will be verified via CO testing. Those with < 10 ppm will be considered abstinent.

#### Secondary outcomes, mediators, and moderators

Study counselors will track counseling data, which will be summarized as “total counseling time” for analyses. We will assess the type, the dose, and the number of days of medication was used via the method of Williams et al. [31, 32]. This will be summarized as “number of days of medication use” for analyses. Default-related variables are derived from the academic literature on choice theory and include smokers’ perceptions of provider attitudes toward tobacco-cessation treatment (implied recommendation), smokers perceptions of the degree to which their provider recommends tobacco-cessation treatment (implied recommendation), perceptions of the “status quo” for hospital tobacco-cessation treatment (status quo bias), and perceptions of paternalistic treatment by UKanQuit staff [13, 23, 33].

#### Intervention costs

We will prospectively track variable intervention costs. Costs will include inpatient counselor services, post-discharge counseling time, and initial pharmacotherapy dispensed at baseline. During the 6-month follow-up call, we will ask participants to recall their use of pharmacotherapy after the initial supply. Personnel time will be valued at Bureau of Labor Statistics (www.bls.gov) wages plus benefits for an appropriately trained health-promotion professional. Pharmacotherapy costs will be based upon retail prices estimated from on-line pharmacy websites. Intervention costs will be tracked as they are incurred. We will exclude research costs. We will not discount either costs or benefits.

#### Fidelity monitoring

Fidelity to the study protocol will be assessed by in-person fidelity assessments during hospital consults. To assess the quality of the intervention and control conditions, we will assess the degree to which study staff accurately: (1) provide brief advice and (2) perform the appropriate intervention for OPT OUT versus OPT IN. Data on fidelity will be entered into a RedCAP database and reported back to study staff to encourage adherence to protocols.

#### Protection against risks

There are minimal risks in this study. Any emails used to transmit study participant information will be encrypted to protect the privacy of patients. Salivary cotinine samples are noninvasive; samples will be labeled with participants’ study ID numbers, rather than names, to protect participants’ privacy. When collecting proxy verification of smoking status, no information about the patients’ participation in the trial will be provided to the proxy besides the participants’ name and the fact that they had nominated the proxy to provide verification of smoking status at the end of a health-promotion study.

Standard language in our consent procedure assures the participants of the confidential nature of the study. Those who elect to participate will be clearly told that they may withdraw from the study at any time without jeopardizing current or future care at any medical facility. Potential participants will also be informed of alternative treatments (i.e., using other smoking-cessation programs, purchasing nicotine gum, patches, or lozenges from the pharmacy, obtaining a prescription for a nicotine inhaler, nasal spray, or other smoking-cessation products from their physician). These standards are strictly adhered to and monitored by the KUMC Institutional Review Board. Only summaries of group data will be reported in any publications or presentations, with no identification of individuals. All records will be kept in locked filing cabinets in offices that are kept locked when unoccupied. Subject files will be kept in a secure area, with access only by designated staff members (PI and co-investigators).

### Data management

Data management will follow procedures developed for Enhancing Quitline Utilization among In-Patients (EQUIP). UKanQuit service data, and survey data collected by research assistants, will be directly entered via tablet into REDCap. The project director will coordinate data retrieval from the EHR. The data manager will conduct initial data cleaning, identifying and tagging any crossovers, conversion into proper format for data analysis, and recoding using standard operating procedures. All data will be imported into SAS for study analyses. Cleaning and management routines (e.g., conversion of birth dates to ages, logical checks for continuous variables, compliance with skip patterns, missing data codes) will be conducted using SAS.

### Data safety and monitoring plan

Due to the low level of risks involved in the proposed study, a Data and Safety Monitoring Board (DSMB) will not be necessary. The data and safety monitoring will be overseen by the PI, Dr. Richter, and an annual progress report will be provided to the Human Subjects Committee of the University of Kansas Medical Center as well as the NIH. The purpose of this data and safety monitoring plan is to ensure the safety of study participants and the validity of data in compliance with National Institutes of Health (NIH) requirement of Data and Safety Monitoring for Clinical Trials. This section outlines essential elements of the Data Safety and Monitoring (DSM) plan for this clinical trial.

#### Plans for assuring data accuracy and protocol compliance

Data management activities for this project will encompass data entry, data cleaning, identifying and tagging any crossovers, conversion into proper format for data analysis and recoding. In addition, a computer-based tracking system will be developed to follow each patient and to prompt the staff for the upcoming data collection point. Data collection points for each subject will be calculated from their initial date of contact. Data entry will be performed on site under direction of the PI and the study statistician. Keypunching routines will adhere to the codebook specifications written by the team. Codebooks will include variable formats (numeric/alpha), min/max ranges and any skip patterns. Data will be double entered utilizing two separate databases and data entry personnel for the two databases would be different. Both the databases and the tracking system would be password protected for security and maintenance of confidentiality. At the end of each data entry period, data would be backed up onto a storage unit. Checks built into the database will ensure that individuals not meeting eligibility criteria are flagged and excluded from data analysis.

#### Data sharing

This study will generate quantitative and qualitative data from the randomized trial. The final quantitative dataset will include self-reported demographic and behavioral data from subjects. Because we collect data at multiple time points from participants over the 6-month period that each participant will be in the study, we will collect identifying information. Prior to data sharing, we will remove or convert all identifying information (date of birth will be converted to age, date of admission and discharge will be removed, and other identifiers will be removed). There may remain the possibility of deductive disclosure of subjects with unusual characteristics. Thus, we will make the data and associated documentation available to users under a data-sharing agreement that provides for: (1) a commitment to using the data only for research purposes and not to identify any individual participant, (2) a commitment to securing the data using appropriate computer technology, and (3) a commitment to destroying or returning the data after analyses are completed. Data will be saved as SAS or SPSS files, saved to disk, and mailed to users.

## Data analysis

### Overview of hypotheses and analyses

**Table 4:** Virtual response patterns for quit rate endpoint

	OPT IN	OPT OUT	
		Efficacy	
No differences	15.7%	15.7%	Both have equal quit rates
Small but unlikely	15.7%	20.0%	OPT OUT is moderately better
Expected	15.7%	25.2%	OPT OUT is better at expected differences

The overall design is a randomized controlled trial (RCT) with individual assignment to groups. Bayesian analysis will drive participant allocation. Prior to outcome analyses we will examine baseline data to evaluate if randomization achieved equivalent groups. In addition, we will conduct process, outcome, mediation, and cost analyses.

#### Bayesian design

We will perform a prospective randomized comparative effectiveness *Bayesian adaptive design study* [34]. Our endpoint is the percentage of patients with verified cessation at 1 month (4 weeks) post randomization. We will perform our first planned interim analyses when we have endpoint data on 400 patients. The arm that appears to be performing the best will get more participants allocated to it in the subsequent randomization period. A new adaptive randomization structure will be updated every 13 weeks, using up-to-date outcome data, until the trial meets success or all 1000 subjects are consented. The main outcome analysis will use an ITT approach, with the denominator being all participants randomized to the study. Data from patients we are unable to reach at 1 month will be de-identified and included in data analyses.

For this study the primary endpoint is modeled:

- *S*_*QjT*_|*n*_*jT*_ ~ Bino(*n*_*jT*_ *,θ* ^*Q*^_*j*_) quitting.

In addition, we provide “weakly informative” priors:

- logit(*θ* ^*Q*^_*j*_) ~ N(0,100^2^).

Using the endpoint data and the prior probabilities, we then use Markov Chain Monte Carlo computations to obtain the Bayesian posterior distributions for the endpoint (i.e., quitting). We will stop randomizing into the comparative trial if the probability of a study arm having maximum utility is greater than 0.9925. The arm having the maximum quit rate is *M*_*T*_ = max(*θ* ^*Q*^_*1,*_ *θ* ^*Q*^_*2*_). The stopping rule is mathematically *P*(*M*_*T*_ > .9925). If a maximum arm is not identified after 500 patients, this procedure and accrual will continue until a maximum arm is identified or we enroll all 1000 patients. It is possible that we will reach the maximum utility criterion before 1000 patients are consented. Should this occur, we will stop the trial and begin outcome, cost, and mediation analyses in order to quickly disseminate findings.

After the maximum arm probability is evaluated, the next round of patients is randomized using a formula that favors the arm with the maximum quit rate, thus taking advantage of the information gained from our interim analyses. Using this formula, each arm is allocated for the next patients to be enrolled in the *j* ^th^ arm proportional to:

$$ {V^{\ast}}_j=\mathrm{sqrt}\Big(\Pr \left({M}_{jT}={\theta^Q}_1\Big)\mathrm{Var}\left({\theta^Q}_1\right)/\left({n}_{jT}+1\right)\right). $$

This type of allocation has more desirable properties than simply using Pr(*M*_*jT*_ = *θ* ^*Q*^_*1*_). In other words, using this approach will allow us to assign more patients to the most promising arm, and fewer patients to the least. Regardless of when the maximum arm probability cut point is reached, we will confirm this finding with a subsequent analysis and evaluation (> .99) after all data from all enrolled patients are obtained, as some will be in the study when early success criteria are identified.

#### Projected quit rates

As defined in the 2012 Cochrane review of smoking cessation in hospitalized patients [35], across both study arms, smokers who receive post-discharge support and medications will receive level-4 intensity treatment and smokers who receive only inpatient brief counseling will receive level-1 intensity treatment. Pooled quit rates are 10% for level-1 intensity studies and 29% for level-4 intensity studies [35]. Based on EQUIP and other trials, we estimate that 30% of OPT-IN participants will be ready to quit and receive services [36, 37]. For OPT OUT, based on the Inter99 trial and our pilot study on proactive quitline counseling we estimate that 80% of patients will accept services [38, 39]. We calculate the proposed quit rates within each condition using the following formula:

$$ \left[\left(\mathit{\mathsf{prop}}.\mathit{\mathsf{receivingservices}}\times \mathit{\mathsf{level}}\ \mathit{\mathsf{4}}\ \mathit{\mathsf{quitrates}}\right)+\left(\mathit{\mathsf{prop}}.\mathit{\mathsf{not}}\ \mathit{\mathsf{receivingservices}}\times \mathit{\mathsf{level}}\ \mathit{\mathsf{1}}\ \mathit{\mathsf{quitrates}}\right)\right]. $$

We estimate the quit rate to be 15.7% in the OPT-IN group: [(.30 × .29) + (.70 × .10)] = .157. We estimate the quit rate to be 25.2% in the OPT-OUT group: [(.80 × .29) + (.20 × .10)] = .252.

#### Power, sample size, and trial duration

In accordance with guidelines for adaptive design power analyses [34], we used simulated data to determine the power, sample size, and anticipated duration of the study. We created virtual responses for our “expected” quit rates, another for “small but unlikely” quit rates, and a third for “no differences” in quit rates. We performed three sets of trial simulations (Table 4). Each set involved many trial simulations that identified power (the probability of success) in two scenarios—one for early success (i.e., being able to stop randomization early) and one for late success (i.e., upon enrolling all 1000 patients). While two of these combinations are very unlikely to occur, we included all scenarios. First, under the “expected” quit rates, we estimated (identified) that 92% of the simulated trials had early success, 3% late success, and only 5% had incomplete results. Thus, this scenario had 95% power. The average sample size of this trial scenario was 625 patients with more than half (387) in the better OPT-OUT arm. The average length of these simulated trials was 98 weeks. Second, if there are “small but unlikely” quit rates, we identified that 30% of the simulated trials had early success, 5% had late success, and 65% had incomplete results. This trial scenario had 35% power and the sample size of this trial scenario was on average 903 with more than half (530) in the better OPT-OUT arm. The average length of this trial scenario was 142 weeks. Third, we examined the scenario that serves as our null hypothesis. In this scenario there are no differences in quit rates among the arms. The extent to which this scenario is “successful” reflects our Type-I error rate. For this scenario, we identified that 4% of the simulated trials had early success, 1% late success. This trial scenario produced an appropriate expected Type-I error (*α* = .05). The sample size of this scenario on average was 986 patients, with about half (495) in the OPT-OUT arm. The average length of the trials under this scenario was 155 weeks—approximately 3 years of recruitment. Hence, our maximal sample size of 1000, in 3 years of recruitment, provides ample time and participants to identify project outcomes under all three scenarios.

**Table 5:** Study hypotheses, measures, and analytic strategy

Purpose	Variables	Analytic strategy
Hypothesis 1: compared to OPT IN, significantly more patients in OPT OUT will participate in counseling, use cessation medications, and be abstinent from smoking	Abstinence: treatment condition and 1-month 7-day point-prevalence abstinence	2-sample binomial test
	Counseling: treatment condition and total counseling time by 1 month	2-sample lognormal test
	Medication: treatment condition and number of days of medication use	2-sample Poisson test
Hypothesis 2: significantly more smokers in OPT OUT will be abstinent from smoking, and mediation analyses will partially or fully explain the effects	Treatment condition and 6-month and • 7-day point-prevalence abstinence • Default variables • Counseling/medication use	Bayesian structural equation modeling with a logistic outcome
Hypothesis 3: OPT OUT will be more costly but also more effective than OPT IN	Treatment condition and 1-month abstinence • Variable costs	Incremental cost/quit

#### Accrual (enrollment) patterns

Accrual patterns refer to how rapidly we enroll patients in the trial. These are important to Bayesian adaptive designs for determining the length of the trial. Based on accrual patterns for EQUIP and other hospital studies conducted by our team, and the sample size required for this trial, we assume that the accrual patterns will follow a Poisson distribution with an average of 6.7 patients per week.

#### Accounting for missing data

For the main outcome analysis, in accordance with the ITT principle, patients lost to follow-up will be considered smokers. We will also calculate main and secondary outcomes using imputed data. Missing observations on covariates will be handled through Bayesian posterior predictive distributions with auxiliary variables, auxiliary variables are variables that are not part of the analysis model, yet are included as missing data correlates to strengthen the assumption that missing covariates are missing at random (MAR; missingness is independent of the missing value) [40–42]. Assuming data are MAR, analyses employed under the ITT principle are considered acceptable.

#### Sensitivity analyses

To explore the effects of assuming MAR when data are actually MNAR (missing not at random) sensitivity analyses will be conducted using either a pattern mixture model or a selection model approach [42–45]. In addition, we will conduct and report an exploratory pooled ITT analysis to examine the effects of the Zelen design by calculating overall quit rates assuming patients who were dropped from the study due to failure to provide consent were (a) smokers, (b) nonsmokers, or (c) MNAR.

### Monitoring the impact of population drift and adaptive randomization

There could be a drift in our population characteristics that are related to outcome [46]. This is a problem for all trials, whether they are equally or adaptively randomized. A recent paper examined the effects of response probabilities that changed over time due to this population drift, and found that population drift had little effect on adaptive randomization [47]. However, given our large dataset, we may be in a unique position to contribute to understanding how adaptive randomization might interact with population drift. In order to determine whether drift occurs, we will monitor the characteristics of the study population over time—especially focusing on drift introduced (or magnified) by adaptive changes in our random allocation rule. We will focus on several factors that are known to be associated with abstinence from smoking. These factors include cigarettes per day, time to first cigarette, sex, age, and income [48]. If we find that sample characteristics become unbalanced, we will adjust our outcome analyses to account for this. In addition, in secondary outcome analyses, we will track 1-month abstinence to determine whether rates changed over time. If we find significant changes in either population characteristics or abstinence over time, we will investigate whether (1) population drift, (2) temporal changes in the randomization scheme, or (3) a combination of the two had an effect on the proportion of patients who quit smoking.

#### Analyses by aim

The main analyses for each aim are outlined below (Table 5).

Aim 1. Hypothesis 1: among patients consented into the trial, significantly more patients in OPT OUT will participate in counseling, use cessation medications, and be abstinent from smoking at 1 month post randomization compared to OPT IN. These analyses will involve two-sample tests.

Aim 2. Hypothesis 2: significantly more smokers in OPT OUT will be abstinent from smoking, and mediation analyses will find that counseling participation, medication use, and default-theory-based variables will partially or fully explain the effect of OPT OUT on cessation at 6 months post randomization. After identifying a structural equation model that adequately fits the data for the hypothesized associations between the intervention and 6-month outcome, i.e., that Comparative Fit Index (CFI) > .9 and Root Mean Square Error of Approximation (RMSEA) < .8, we will examine mediation using approaches based on the work of MacKinnon et al. [49], Brown [50], and Shrout and Bolger [51]. Our theoretical model (Fig. 1, Significance) will guide our analysis.

Aim 2. Hypothesis 3: OPT OUT will be more costly—in terms of upfront costs—but will be more effective than OPT IN. As a result, OPT OUT will be more cost-effective from a provider perspective. We will conduct a cost-effectiveness analysis to explicitly document the relative costs and benefits of OPT OUT versus OPT IN. Our cost analytic framework generally follows the guidelines adopted by the Centers for Disease Control (CDC) in accordance with the Consensus Panel on Cost-effectiveness in Health and Medicine [52–54]. We will divide the analysis into two components: first, intervention-only costs, and second, intervention plus short-term (6 months or less) costs post discharge. The primary cost-effectiveness analysis will be set up as an incremental cost-effectiveness ratio (ICER). Incremental cost-effectiveness analysis identifies the marginal benefit of switching from one intervention to the other and is the ratio of the difference in costs divided by the difference in effectiveness between the two study arms. The outcome assessed will be biochemically or proxy-verified 7-day point-prevalence abstinence. The ICER will indicate the added cost per additional quitter OPT OUT versus OPT IN, a metric that will allow comparisons to other smoking-cessation economic studies. While the societal perspective is recommended by current national guidelines, it requires quality-adjusted life years (QALYs) as the denominator [52]. Since this is a short-term study, we decided against attempting to estimate changes in QALYs, and focus instead on cost per quit and incorporate costs from a health care system perspective (direct costs only), comparable to a-priori cost analyses of several clinical trials based in Kansas [55, 56]. In sensitivity analyses, we will adjust wage rates upwards to the national average. In order be able to generalize our findings from this one clinical trial to other populations, we will explore how the variation in counseling time and effectiveness influence the relative cost-effectiveness of the treatment strategies. Our analyses will vary time and effectiveness until breakeven points are achieved between the treatment options.

## Discussion

### Research contribution

Missed opportunities for providing medications and counseling for tobacco dependence has created a large treatment gap. This study innovatively reframes the rationale for this gap: what if low rates of treatment are caused *not* by smokers’ lack of motivation to quit, but by requiring patients to opt in to care? Viewed from this perspective, the effects of screening for readiness on tobacco-cessation treatment can be tested by changing the way that treatment is offered. This study also identifies a major rate-limiting step in access to tobacco-cessation treatment. Regardless of what population, setting, or approach is used to disseminate tobacco-cessation treatment, as long as providers screen for readiness, the majority of smokers will report that they are *not* ready to quit—and that they will *not* receive medications or counseling.

The modified Zelen design is a novel feature of this trial, which will permit a true population-based study in which all smokers, regardless of their level of motivation, will be included in the study. By consenting patients after random assignment and treatment, the study does run the risk of dilution bias introduced by participants refusing consent post randomization [22]. A recent comprehensive review of Zelen trials found that most trials experience low dilution bias (single-digit percentages of refusal to consent or crossover). This suggests that the potential for recruiting a more representative and generalizable sample greatly outweighs the dilution bias introduced at the time of consent.

The Bayesian adaptive design approach is another novel feature of this trial. It is a highly efficient and ethical strategy for comparative effectiveness clinical trial design because it allocates more patients to effective treatments and can answer the research question earlier than conventional designs [57, 58]. In this approach one primary endpoint is used to drive the adaptive randomization. This endpoint is compared across study groups periodically, and more patients are randomized to the stronger arm until a predetermined probability that one arm has “maximum utility” is reached, which signals the end of the comparative trial.

This trial will identify the impact of default tobacco-cessation treatment, for an entire population of smokers, on (1) use of medications and counseling and (2) cessation. It will identify the costs and cost-effectiveness of changing the default, and examine psychological mediators of change based on decision theory. This contribution is significant because it will definitively determine the impact of routinely assessing smokers’ readiness to quit in real-world clinical practice. Assessing readiness is an integral step in the “5 As” of tobacco-cessation treatment (*Ask, Advise, Assess* [*readiness to quit*]*, Assist, Arrange*) [3]. But the *Assess* step is only supported by “C-level” evidence of effectiveness, as there are no trials that establish that it has a positive impact on cessation [3]. Indeed, we hypothesize that the *Assess* step may actually reduce the number of smokers that receive evidence-based treatment. If our hypothesis is confirmed, it could transform the “5 A” approach and increase access to treatment for the more than 30 million smokers that visit a health care provider each year [59, 60].

## Trial status

Recruitment of participants started on 12 September 2016 and at the time of submission of this manuscript, enrollment is ongoing (Additional file 1).

## Additional file

Additional file 1: SPIRIT 2013 Checklist: recommended items to address in a clinical trial protocol and related documents. (DOC 122 kb)

## Abbreviations

CFI: Comparative Fit Index
DSM: Data Safety and Monitoring
DSMB: Data and Safety Monitoring Board
EHR: Electronic health record
EQUIP: Enhancing Quitline Utilization among In-Patients
ICER: Incremental cost-effectiveness ratio
IRB: Institution Review Board
ITT: Intent-to-treat
KUMC: University of Kansas Medical Center
MAR: Missing at random
MNAR: Missing not at random
NIH: National Institutes of Health
NRT: Nicotine Replacement Therapy
PI: Principal investigator
QALYs: Quality-adjusted life years
RCT: Randomized controlled trial
RMSEA: Root Mean Square Error of Approximation
SAS: Statistical Analysis System
SPSS: Statistical Package for the Social Sciences
STEMI: ST-elevation myocardial infarction
UKanQuit: University of Kansas Hospital Smoking Cessation Service
